# Definition and Criteria for Diagnosing Cesarean Scar Disorder

**DOI:** 10.1001/jamanetworkopen.2023.5321

**Published:** 2023-03-29

**Authors:** Saskia J. M. Klein Meuleman, Ally Murji, Thierry van den Bosch, Oliver Donnez, Grigoris Grimbizis, Ertan Saridogan, Frederick Chantraine, Tom Bourne, Dirk Timmerman, Judith A. F. Huirne, Robert A. de Leeuw

**Affiliations:** 1 Amsterdam University Medical Center, Vrije Universiteit Amsterdam, Department of Obstetrics and Gynaecology, De Boelelaan 1117, Amsterdam, the Netherlands; 2 Amsterdam Reproduction and Development, Amsterdam, the Netherlands; 3 Department of Obstetrics and Gynaecology, University of Toronto, Mount Sinai Hospital, Toronto, Ontario, Canada; 4 Department of Obstetrics and Gynecology, University Hospital KU Leuven, Herestraat 49, 3000, Leuven, Belgium; 5 Department of Development and Regeneration, KU Leuven, Belgium; 6 Complex Endometriosis Center. Polyclinique Urbain V, (Elsan Group), Avignon, France; 7 1st Department of Obstetrics and Gynaecology, Medical School, Aristotle University of Thessaloniki, Greece; 8 Reproductive Medicine Unit, Elizabeth Garrett Anderson Wing Institute for Women’s Health, University College Hospital, NW1 2BU London, United Kingdom; 9 Department of Obstetrics and Gynecology, Hopital Citadelle, CHU Liege, Liege, Belgium; 10 Institute for Reproductive and Developmental Biology, Imperial College, London, United Kingdom

## Abstract

**Importance:**

Approximately 60% of women develop a uterine niche after a cesarean delivery (CD). A niche is associated with various gynecological symptoms including abnormal uterine bleeding, pain, and infertility, but there is little consensus in the literature on the distinction between the sonographic finding of a niche and the constellation of associated symptoms.

**Objective:**

To achieve consensus on defining the clinical condition that constitutes a symptomatic uterine niche and agree upon diagnostic criteria and uniform nomenclature for this condition.

**Design, Setting, and Participants:**

A consensus based modified electronic Delphi (eDelphi) study, with a predefined Rate of Agreement (RoA) of 70% or higher. Experts were selected according to their expertise with niche-related consultations, publications, and participation in expert groups and received online questionnaires between November 2021 and May 2022.

**Main Outcomes and Measures:**

Definition, nomenclature, symptoms, conditions to exclude, and diagnostic criteria of an illness caused by a symptomatic uterine niche.

**Results:**

In total, 31 of the 60 invited experts (51.7%) participated, of whom the majority worked in university-affiliated hospitals (28 of 31 [90.3%]), specialized in benign gynecology (20 of 31 [64.5%]), and worked in Europe (24 of 31 [77.4%]). Three rounds were required to achieve consensus on all items. All participants underlined the relevance of a new term for a condition caused by a symptomatic niche and its differentiation from a sonographic finding only. Experts agreed to name this condition cesarean scar disorder, defined as a uterine niche in combination with at least 1 primary or 2 secondary symptoms (RoA, 77.8%). Defined primary symptoms were postmenstrual spotting, pain during uterine bleeding, technical issues with catheter insertion during embryo transfer, and secondary unexplained infertility combined with intrauterine fluid. Secondary symptoms were dyspareunia, abnormal vaginal discharge, chronic pelvic pain, avoiding sexual intercourse, odor associated with abnormal blood loss, secondary unexplained infertility, secondary infertility despite assisted reproductive technology, negative self-image, and discomfort during participation in leisure activities. Consensus was also achieved on certain criteria that should be met and conditions that should be excluded before making the diagnosis.

**Conclusions and Relevance:**

In this modified Delphi study, a panel of 31 international niche experts reached consensus for the constellation of symptoms secondary to a uterine niche and named it cesarean scar disorder.

## Key Points

**Question:**

How do experts define the clinical condition that constitutes a symptomatic niche in the uterine cesarean scar?

**Findings:**

In this modified Delphi study, which included 31 international gynecologists, consensus was achieved on the definition, symptoms, conditions to exclude, and diagnostic criteria of a cesarean scar disorder, following a modified Delphi procedure.

**Meaning:**

Using a standardized definition for the constellation of symptoms resulting from a symptomatic uterine cesarean scar will allow better recognition of this condition, improve patient care, prevent overtreatment, and create a patient-centric foundation of niche-related research in the future.

## Teaser

This qualitative study evaluates how experts define and diagnose cesarean scar disorder.

## Introduction

Cesarean delivery (CD) is the most commonly performed surgery worldwide.^1^ A long-term complication of a CD is an unhealed defect in the uterine myometrium, which is often referred to as a uterine niche or cesarean scar defect. This entity is formally defined by the European Niche Taskforce as an “indentation in the uterine myometrium of at least 2 mm at the site of the cesarean scar assessed by transvaginal ultrasound.”^2^ A niche is observed in 60% of women after a CD and 25% of all women have a large defect with a residual myometrium of less than 3 mm.^3,4^ Approximately 30% to 40% of women with a CD niche experience symptoms^5^ such as postmenstrual spotting, dysmenorrhea, chronic pelvic pain, and infertility.^3,6,7,8^ Symptomatic niches can have a profound impact on patients’ quality of life.^9^

Given that the CD niche is a fairly new but increasingly common observed entity, the literature reports a wide variety of symptoms, conditions, and therapies for patients with a niche.^10,11,12^ Studies include heterogenous patient populations from asymptomatic to those with various symptom profiles, where the association with the niche may be tenuous. Consequently, due to lack of clear guidelines, it is difficult to propose optimal treatments and informed counseling to patients with a niche. It is essential that future clinical practice guidelines and policies make the distinction between a sonographic finding of a niche and the constellation of associated symptoms. The objective of this electronic Delphi (eDelphi) study was to reach consensus among international experts on defining the clinical condition that constitutes a symptomatic niche in the uterine CD scar and agree upon diagnostic criteria and uniform nomenclature for this condition.

## Methods

We performed an eDelphi study between November 22, 2021, and May 16, 2022. A Delphi is an iterative process with anonymous consultation, feedback, and qualitative analysis of the responses. This technique has been used widely in health care research, within the field of education, and in developing clinical practice.^13,14^ To this end, we designed an electronic platform to efficiently obtain consensus from international experts. The purpose of this design was to continue with rounds until consensus was achieved for all questions. To determine the first round of questions, we performed a systematic review of the available evidence, followed by a focus group discussion with experts. After formulating the questionnaire, we assembled the Delphi panel and started the first round. Approval of this study was granted by the institutional review board of Amsterdam University Medical Center (location VUmc) and registered at Open Science Framework registries. Participants provided written informed consent. The Standards for Reporting Qualitative Research (SRQR) and Preferred Reporting Items for Systematic Reviews and Meta-analyses (PRISMA) reporting guidelines guided the study process.^15^

### Systematic Literature Review and Focus Group Discussion

A systematic literature search was performed to evaluate symptoms that are reported in association with a niche. We used the previously published systematic review of Stegwee et al^9^ and updated this search in November 2021 (the search strategy and results are presented in the eAppendix and eFigure in Supplement 1). We included full-text English language studies that reported on symptoms associated with CD niche. These studies were evaluated by 2 independent researchers (S.K. and R.L.) who extracted baseline characteristics of the studies, methods, reported symptoms, and their prevalence. According to this literature review, a focus group of 5 Dutch niche experts helped determine the list of symptoms and questions to be included in the first round of the eDelphi. These experts evaluate at least 50 patients with niche-related issues annually. The first round consisted of items categorized into the following 6 themes: (1) nomenclature, (2) gynecological symptoms, (3) fertility-related symptoms, (4) social relationships and participation, (5) obstetrical symptoms, and (6) conditions to exclude and diagnostic criteria.

### Expert Panel Recruitment

We defined a niche expert as an obstetrician or gynecologist who consults on more than 30 niche-related issues annually and has participated or is actively participating in niche or CD-related research. Potential experts were identified through our literature search and society membership (European Niche Taskforce, Cesarean Scar Pregnancy Registry Team Steering Group, and International Society for Placenta Accreta Spectrum). Potential eligible experts were invited to complete a questionnaire to determine eligibility and were subsequently invited to participate if they met inclusion criteria.

### Patient Representative Endorsement

It was important for us to incorporate the patient perspective in this process. The questions of the first round of the Delphi were based on a focus group discussions among patients, which has been published separately.^9^ Finally, after the Delphi was completed, we performed a focus interview with experienced representatives to discuss the results. The patients who were involved were recruited from Amsterdam University Medical Centre or were representatives of the Dutch niche Facebook group.^16^ Those with a large symptomatic niche were invited to participate in an online meeting to discuss the proposed definition, symptoms, conditions to exclude, and diagnostic criteria. Women who volunteered to participate signed an informed consent.

### Statistical Analysis

#### eDelphi Rounds 1 to 3

All questions in the first round (see eTable in Supplement 1) were accompanied by an overview of the available literature. In the survey, participants were asked to evaluate each statement using a 3-point Likert scale (agree, neutral, or disagree), or indicate “not my expertise.” We used the following definitions: agreement: “I agree with the following statement;” disagreement: “I do not agree with the following statement;” neutral: “I do not have an opinion about the following statement.” These definitions are consistent with previously published work.^17,18^ All survey items included space for comments and reflections. In subsequent rounds, an overview of the results of the previous round was provided. When consensus was not reached for a particular concept, the arguments of all experts were anonymously shown in the next round for participant consideration. This allowed us to emulate the spirit of discussion that is essential in Delphi consensus such that anonymized arguments could be shared between the experts. The questions that did not reach consensus were then either repeated or rephrased according to the input given. Additional clarifying questions were included as required. The Figure shows the flow diagram showing agreement or rejection of items during the Delphi procedure.

**Figure.**
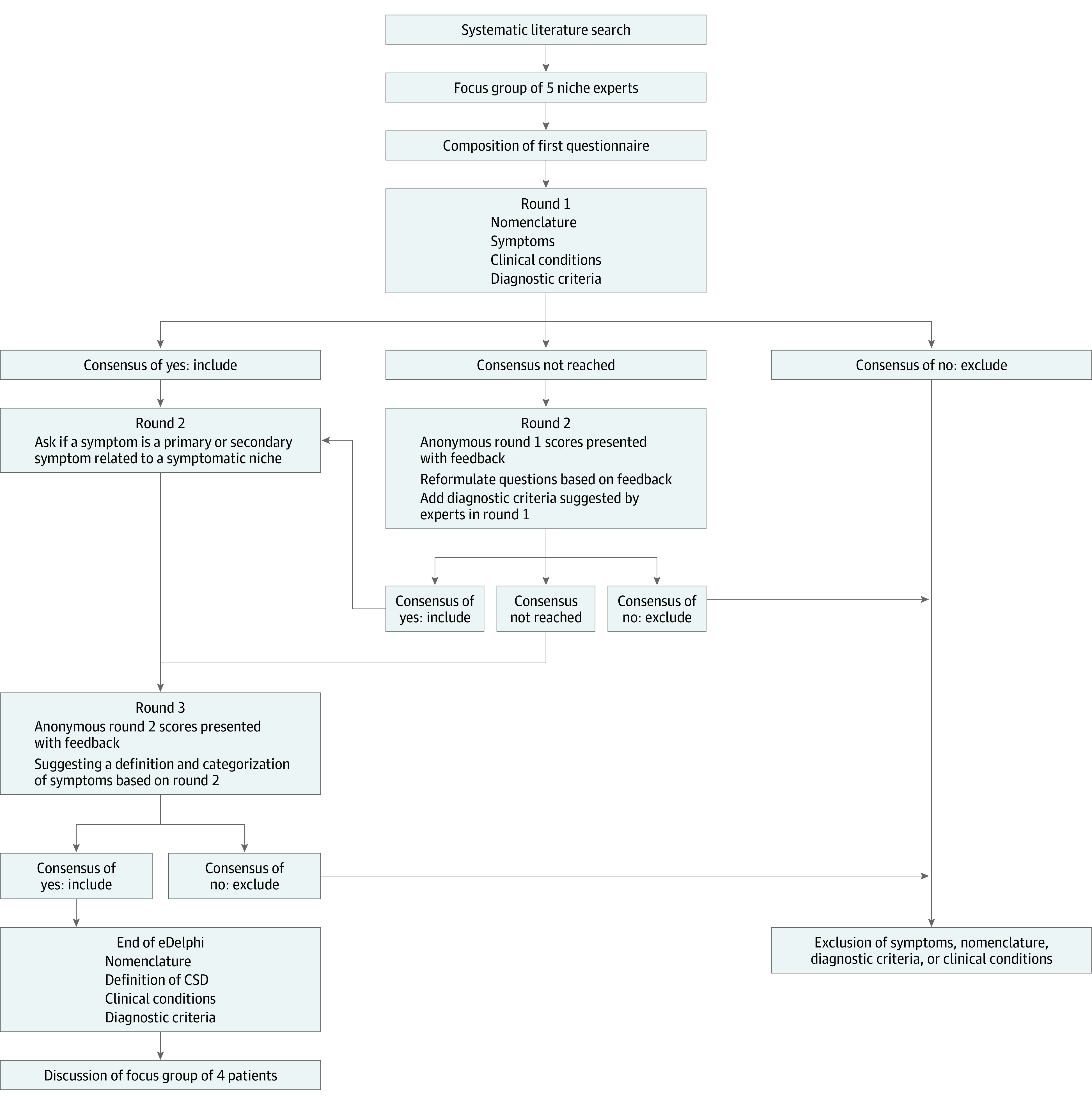
Flow Diagram Summarizing Agreement or Rejection of Items During Delphi Procedure Items were accepted if consensus agreement of at least 70% was reached.

#### Definition of Consensus

Consensus was predefined, in keeping with previously published work,^17,18,19^ as a Rate of Agreement (RoA) of 70% or greater, where RoA = (agreement – disagreement)/ (agreement + disagreement + neutral) × 100%. The experts who selected “not my expertise” were not included in the RoA calculation for that question. All analyses were done in SPSS version 28 (IBM).

## Results

Among the 31 experts who met the inclusion criteria (51.7%), the majority worked in university-affiliated hospitals (28 of 31 participants [90.3%]). Participating experts represented 3 continents and obstetrics (7 of 31 participants [22.6%]), benign gynecology (20 of 31 participants [64.5%]) and fertility (4 of 31 participants [12.9%]) subspecialties. In total, 29 (93.5%) experts completed all rounds. Originally, we invited 57 potential experts to participate; 3 experts were subsequently invited following recommendation by their colleagues. Baseline characteristics are presented in Table 1. A total of 20 of 21 items of the SRQR checklist were met.

**Table 1.** Demographic Characteristics of Experts Who Responded to the Questionnaires

Characteristic	Experts responded in rounds 1 and 2, No. (%) (n = 31)	Experts responded in round 3, No. (%) (n = 29)
Primary location		
Europe	24 (77.4)	22 (75.9)
North America	2 (6.5)	2 (6.9)
Asia	5 (16.1)	5 (17.2)
Primary subspecialization		
Benign gynecology	20 (64.5)	19 (65.5)
Fertility	4 (12.9)	4 (13.8)
Obstetrics	7 (22.6)	6 (20.7)
Main level of care		
Private clinic	1 (3.2)	1 (3.4)
Referral center for niche related problems	2 (6.5)	2 (6.9)
University hospital	28 (90.3)	26 (89.7)
Niche operations performed a year		
0-15	6 (19.4)	5 (17.2)
16-30	2 (6.5)	1 (3.4)
31-50	6 (19.4)	6 (20.7)
>50	9 (29.0)	9 (31.0)
Not applicable	8 (25.8)	8 (27.6)

### eDelphi Rounds 1 to 3

In the first round, experts evaluated 29 symptoms potentially associated with a niche, which were identified by the literature search and suggested by the focus group. The process is shown in the Figure. They also assessed 5 potential diagnostic criteria, 5 potential conditions requiring exclusion before a niche could be diagnosed, and 2 questions about niche nomenclature. All experts participated in the first and second rounds. Twenty-nine experts (93.5%) participated in the third round, after which consensus was reached for the classification on all symptoms and nomenclature. Detailed round-by-round consensus results can be found in the eTable in Supplement 1.

### Relevance of the Disorder

There was consensus on the statement that “it is important to differentiate between a sonographic finding of a niche and a condition caused by a niche-related symptoms and the effect on quality of life” (RoA 100%). There was also consensus that the sonographic finding should be defined and evaluated as previously published by Jordans et al^2^ (ie, an indentation at the site of the CD scar with a depth of at least 2 mm) (RoA 77.8%).

### Nomenclature

The preferred term for a niche that caused symptoms was Cesarean Scar Disorder (CSDi) (RoA 96.2%). Other options that were discussed included niche disorder, niche disease, cesarean scar disease and cesarean scar syndrome. The most salient argument that led experts to choose CSDi as the preferred term was that the name implied the origin of the symptoms and emphasized that as an abnormal condition.

### Symptoms

During the eDelphi rounds, the desire to classify the symptoms related to a niche emerged. After completion of the final round, consensus was reached (RoA 77.8%) defining a CSDi as a condition that includes at least 1 primary or 2 secondary symptoms in combination with a sonographic finding of a niche, according to the definition by Jordans et al.^2^ Table 2 outlines the primary and secondary niche-related symptoms, with the corresponding RoA.

**Table 2.** Consensus-Based Definition of Primary and Secondary Symptoms of Cesarean Scar Disorder

Primary symptom/problems	RoA, %	Secondary symptoms	RoA, %
Postmenstrual spotting	80.6	Dyspareunia	72.7
Pain during uterine bleeding	84.2	Abnormal vaginal discharge	87.5
Technical issues with catheter insertion during embryo transfer	73.1	Chronic pelvic pain	87.1
Secondary unexplained infertility combined with intrauterine fluid	70.4	Avoiding sexual intercourse	90.3
NA	NA	Odor associated with abnormal blood loss	83.4
NA	NA	Secondary unexplained infertility	70.0
NA	NA	Secondary infertility despite ART	72.4
NA	NA	Negative self-image	88.0
NA	NA	Discomfort during participation in leisure activities	77.3

Abbreviations: ART, assisted reproductive technology; NA, not applicable; RoA, rate of agreement.

The experts concluded that obstetrical issues related to a niche, such as a Cesarean Scar Pregnancy, uterine dehiscence/rupture, or placenta accreta spectrum should be reported as complications of the CSDi (RoA 70.4%) and should not be classified as a primary or secondary symptom. Experts agreed that there was insufficient literature to determine whether a miscarriage in patients with a niche should be classified as a symptom related to the CSDi or that it should be considered as an independent problem with a different cause (RoA 96.7%). This was identified as a relevant knowledge gap that requires additional research before it can be considered in future updates of the CSDi definition, which should be reevaluated after 1 year.

### Diagnostic Criteria and Conditions to Exclude

Before a symptom-based diagnosis of CSDi can be made, experts agreed that certain minimum criteria should be met. These included a minimum of 3 regular menstrual cycles after a CD before diagnosis (RoA 93.5%); a patient needing to be premenopausal (RoA 100%); and the complaints of a symptomatic niche starting after a CD or significantly worsening after a CD (RoA 80.6%). Consensus was also reached about a statement that a patient can be cured from a symptomatic niche, however this does not mean that all symptomatic niches should be treated (RoA 87.1%). Experts also felt that before confirming a diagnosis of CSDi, certain conditions should be excluded, including cervical dysplasia (RoA 74.2%), vaginal or uterine infections (RoA 74.2%), other uterine intracavitary pathology (RoA 93.5%), anovulatory cycles (RoA 100%), or other causes of postmenstrual spotting (RoA 80.6%) (Table 3).

**Table 3.** Criteria Required for Diagnosis of Cesarean Scar Disorder and Conditions That Should Be Ruled Out Before Confirming the Diagnosis

Diagnostic criteria	RoA, %	Conditions to exclude	RoA, %
Minimum of 3 regular menstrual cycles after CD before diagnosis can be made	93.5	Cervical dysplasia	74.2
A patient needs to be premenopausal	100	Vaginal/uterine infections	74.2
The complaints of a symptomatic niche should start after a CD or should worsen significantly after a CD	80.6	Other uterine intracavitary pathology	93.5
A patient can be cured from a symptomatic niche (this does not mean that all symptomatic niche should be treated)	87.1	Other causes of postmenstrual spotting (such as continuous oral contraceptive use or intrauterine device)	80.6
NA	NA	Anovulatory cycles	100

Abbreviations: CD, cesarean delivery; NA, not applicable; RoA, rate of agreement.

### Patient Representative Endorsement

In total 4 patient representatives participated in the online meeting to provide input on the relevance, clarity of the consensus statements, and suggested possible missing items from a patients’ perspective. All patient representatives agreed with the proposed definition and suggested no additional terms from their perspective. They approved the intended revision after 1 year. They underlined the need for clear diagnostic criteria and urged the development of clinical guidelines on diagnosis and therapy to guide clinicians in the management of CSDi.

## Discussion

### Main Findings

In this modified Delphi study, international experts specializing in the management of CD niche agreed that the constellation of symptoms resulting from a CD niche should be termed Cesarean Scar Disorder (CSDi). The agreed definition for CSDi was at least 1 primary or 2 secondary symptoms in the presence of a sonographic finding of a niche, according to the definition by Jordans et al^2^ (ie, an indentation at the site of the CD scar with a depth of at least 2 mm). CSDi is a condition that affects premenopausal women who are symptomatic for at least 3 months following their CD. This definition helps to discriminate between a sonographic finding and a relevant condition that impacts quality of life. Experts also emphasized that before a diagnosis of CSDi is made, certain conditions must be excluded (eg, cervical dysplasia, infection, uterine cavity pathology, or abnormal uterine bleeding from ovulatory or other iatrogenic causes).

### Clinical Implications

The ability to diagnose CSDi is of great clinical value. It is important to state that a diagnosis of CSDi does not necessarily mean that treatment is indicated. The decision to investigate and treat CSDi is influenced by the symptom burden, impact on quality of life, size of the niche, and the individualized expectations of the patient.

This consensus by international experts and patients to formally recognize a disorder caused by a CD niche creates for the first time a medical condition, with specific diagnostic and exclusion criteria. This will have significant ramifications for various groups. First, it will allow patients to be recognized and their symptoms to be taken seriously. It will also enable them to advocate for themselves as we already know the burden that niche-related symptoms can have on patients’ emotional, physical, and social quality of life.^8^ Second, by formally creating and recognizing a specific disorder, along with associated inclusion and exclusion criteria, it provides an opportunity for frontline women’s health physicians to be educated about this issue and consequently improve the care they provide for these patients. Lastly, this newly agreed upon entity of CSDi and its clear definition will facilitate standardization of care and niche-related research. It will therefore be useful for the development of future systematic reviews, meta-analyses and guidelines. Hopefully these all will prevent both the over- and undertreatment of CD niches.

Although surgical management of CSDi has been investigated in an RCT for the amelioration of abnormal uterine bleeding,^20^ data for treating other niche-related symptoms is very scarce.^21^ Currently 1 RCT^22^ has been registered that compares the effect of a laparoscopic niche resection vs expectant management in infertile women with a large niche, and an RCT^23^ that evaluates the effect of a laparoscopic niche resection compared with hormonal treatment in women with niche-related spotting complaints is underway as well. With respect to hysteroscopic niche resection, 1 study evaluated the effect on clinical pregnancy rate in 61 secondary infertile patients and showed superiority of the hysteroscopic niche resection compared with expectant management.^24^ As part of good clinical practice, other conditions should be considered as a part of differential diagnosis dependent on the patients’ symptoms, such as the presence of adenomyosis or deep endometriosis in women with dysmenorrhea. Additionally, imaging, dynamic ultrasound,^25^ or MRI can help to differentiate between these disorders.

A CSDi is an iatrogenic consequence of a CD. However, current international guidelines on Cesarean deliveries do not include information on the potential development of niche-related symptoms such as postmenstrual spotting or niche-related fertility problems after a CD.^26,27^ Given the high incidence of these problems and the acknowledged relevance by both patients and experts, we propose to inform patients on the possibility of developing CSDi after a CD. In addition, more attention should be given by clinicians to women who report these symptoms after a CD. The agreed upon nomenclature, definitions, and criteria for CSDi will increase awareness of these problems both by women and clinicians. Furthermore, as discussed before, it may also contribute to the development of evidence-based guidelines that may prevent both over- and under treatment.

### Comparison to Other Literature

Although the number of published studies on CD niche–related issues and applied therapies has increased over the last decade, to the best of our knowledge, there is no uniform, international definition of a disorder caused by a symptomatic niche. In the literature different terms are used, such as isthmocele,^28^ cesarean scar syndrome,^29^ cesarean scar defect,^6,30^ or even CD disorder.^31^ However, none of the studies reported a clear definition or diagnostic criteria.

The results of this study are intended to guide appropriate clinical decision-making and the unambiguous inclusion of women in niche-related research. However, using 1 primary or 2 secondary symptoms as diagnostic criteria has not been clinically tested. We realize that there is currently no information on the weight of individual symptoms or classification of their clinical implications. We would like to stress that the proposed definition of CSDi and related diagnostic criteria are only a starting point and that the relevance of the number and weight of these symptoms should be assessed in future studies. This is in line with the development of the definitions of many other disorders. As an example, current polycystic ovary syndrome (PCOS) criteria evolved over many years with the diagnosis changing over time as new information became available.^32^

### Strengths and Limitations

The most important strength of our study is the use of a Delphi technique. This procedure allows multiple experts to discuss and consent to complex questions, with the assurance of anonymity and the benefit of equal input in the final consensus statement. Another strength was the rigorous systematic review of the literature that guided the creation of the first questionnaire and to guide experts in the first Delphi round.^9^ Additionally, the expert panel was diverse in subspecialization, country of origin, and expertise.

Our results should be interpreted in the context of the study design. Although we tried to define preselected criteria, we appreciate that there will be a selection bias according to the experts approached to participate and that not all countries are represented. Furthermore, in the focus group to design the first questionnaire, only Dutch experts participated, which may have influenced the content of the first questionnaire. For this reason, international experts were encouraged to add free text in each round on topics or items that may have been missed. Another consideration with our findings is that due to the novelty of this subject there is an exponential growth of literature on this topic, with addition of new findings continuously. For these reasons, we encourage this definition to be revisited regularly in a context that includes more experts and incorporates the latest data and findings on the topic with validation using diverse patient groups. The ethno-cultural impact of niche-related symptoms is an important consideration as the definition of CSDi is applied worldwide.

## Conclusion

In this modified Delphi study, criteria for a cesarean scar disorder were defined as a condition with at least 1 primary or 2 secondary symptoms in association with a niche in the uterine CD scar. Our study should be considered a good starting point that aims to develop future guidelines to give women with a CSDi the recognition and care they need.

## Notes

Supplement 1. **eAppendix. **Search Strategy **eFigure. **Flowchart Showing Studies Identified Through Literature Search **eTable. **Results of Consensus Rounds

Supplement 2. **Nonauthor Collaborators. **The CSDi Study Group

Supplement 3.
Data Sharing Statement
