# Supplementary material for: Definition and Criteria for Diagnosing Cesarean Scar Disorder
Source: JAMA Netw Open. 2023 Mar 29;6(3):e235321. doi: 10.1001/jamanetworkopen.2023.5321 (PMC10061236; doi:10.1001/jamanetworkopen.2023.5321)
Supplement: Supplement 1. — eAppendix. Search Strategy eFigure. Flowchart Showing Studies Identified Through Literature Search eTable. Results of Consensus Rounds [file jamanetwopen-e235321-s001.pdf]

Supplemental Online Content

Klein Meuleman SJM, Murji A, van den Bosch T, et al; CSDi Study Group. Definition and criteria for diagnosing cesarean scar disorder. *JAMA Netw Open*. 2023;6(3):e235321. doi:10.1001/jamanetworkopen.2023.5321

eAppendix. Search Strategy

eFigure. Flowchart Showing Studies Identified Through Literature Search

eTable. Results of Consensus Rounds

This supplemental material has been provided by the authors to give readers additional information about their work.

© 2023 Klein Meuleman SJM et al. *JAMA Network Open.*

eAppendix 1. Search strategy

PubMed 5 November 2021

Search	Query	Results
#4	#1 AND #2 AND #3	454
#3	("Cesarean Section"[MeSH Terms] OR "cesarea*"[Title/Abstract] OR "caesarea*"[Title/Abstract] OR "c section"[Title/Abstract] OR "c sections"[Title/Abstract] OR ("abdominal"[Title/Abstract] AND "deliver*"[Title/Abstract]) OR "postcesarea*"[Title/Abstract] OR "postcaesaria*"[Title/Abstract]) AND (2019/6/21:2021/11/5[pdat])	11.199
#2	("Uterus"[MeSH Terms] OR "Uterine Diseases"[MeSH Terms] OR "Uterus"[Title/Abstract] OR "uterine"[Title/Abstract] OR "myometri*"[Title/Abstract] OR "endometri*"[Title/Abstract] OR "endomyometri*"[Title/Abstract] OR "myoendometri*"[Title/Abstract]) AND (2019/6/21:2021/11/5[pdat])	32617
#1	("Cicatrix"[MeSH Terms] OR "cicatr*"[Title/Abstract] OR "scar"[Title/Abstract] OR "scars"[Title/Abstract] OR "scarring"[Title/Abstract] OR "isthmocele*"[Title/Abstract] OR "niche"[Title/Abstract] OR "niches"[Title/Abstract] OR "anechoic"[Title/Abstract] OR "pouch*"[Title/Abstract] OR "diverticul*"[Title/Abstract]) AND (2019/6/21:2021/11/5[pdat])	28399

Embase 5 November 2021

Search	Query	Results
#5	#1 AND #2 AND #3 AND [embase]/lim	771
#4	#1 AND #2 AND #3	826
#3	('cesarean section'/exp OR cesarea*:ab,ti OR caesarea*:ab,ti OR 'c section':ab,ti OR 'c sections':ab,ti OR (abdominal:ab,ti AND deliver*:ab,ti) OR postcesarea*:ab,ti OR postcaesarea*:ab,ti) AND [21-06-2019]/sd NOT [06-11-2021]/sd	23589
#2	('uterus'/exp OR 'uterus disease'/exp OR uterus:ab,ti OR uterine:ab,ti OR myometri*:ab,ti OR endometri*:ab,ti OR endomyometri*:ab,ti OR myoendometri*:ab,ti) AND [21-06-2019]/sd NOT [06-11-2021]/sd	61054
#1	('wound dehiscence'/exp OR 'scar formation'/exp OR 'scar'/exp OR cicatr*:ab,ti OR scar:ab,ti OR scars:ab,ti OR scarring:ab,ti OR isthmocele*:ab,ti OR niche:ab,ti OR niches:ab,ti OR anechoic:ab,ti OR pouch*:ab,ti OR diverticul*:ab,ti) AND [21-06-2019]/sd NOT [06-11-2021]/sd	45232

© 2023 Klein Meuleman SJM et al. *JAMA Network Open.*

eFigure 1. Flowchart Showing Studies Identified Through Literature Search

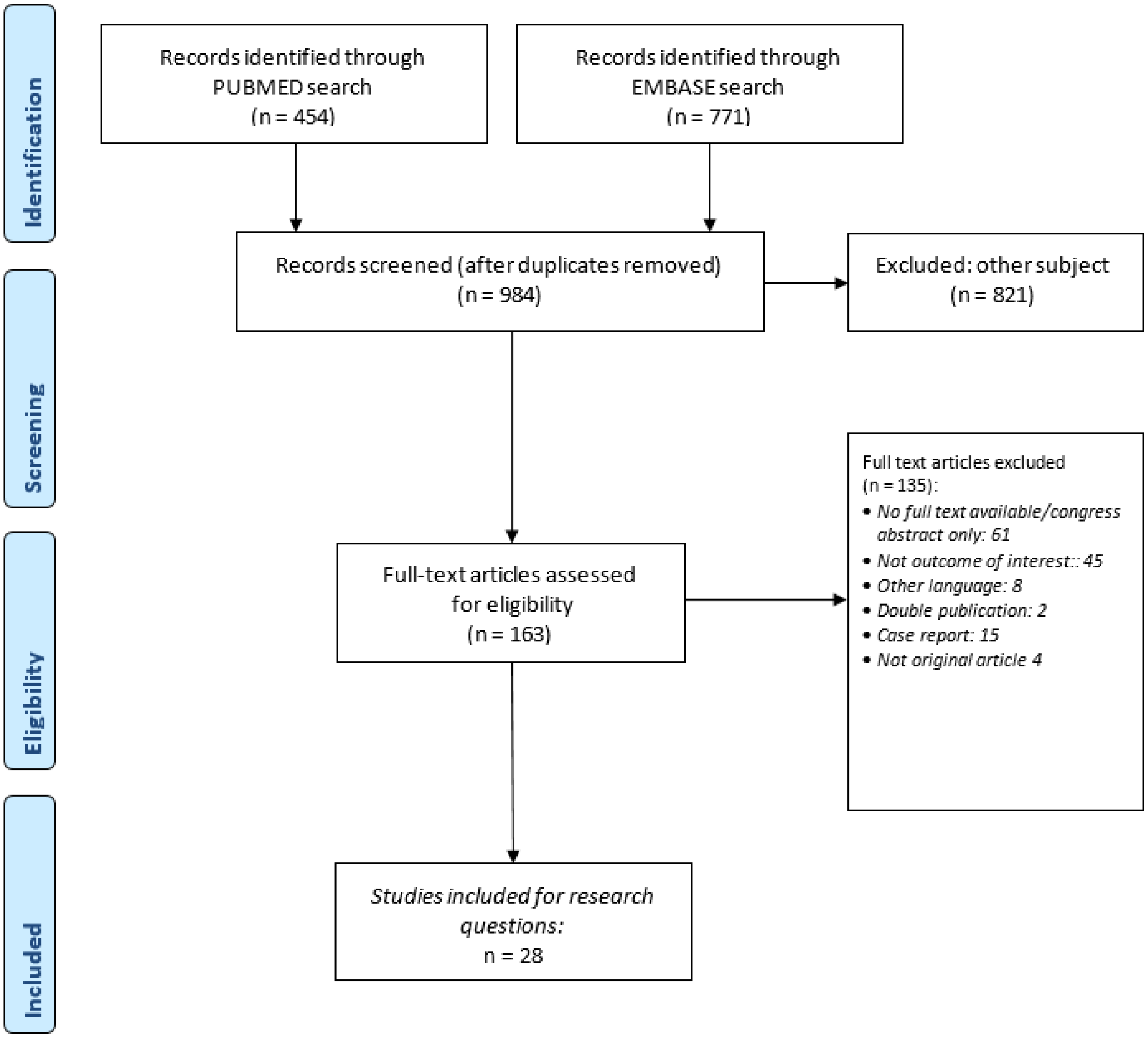

© 2023 Klein Meuleman SJM et al. *JAMA Network Open.*

eTable 1. Results of Consensus Rounds

Nomenclature

Question	RoA in round 1(%)	Consensus reached in round 1	RoA in round 2 (%)	Consensus reached in round 2	RoA in round 3 (%)	Consensus reached in round 3
Which of the following conditions would best cover the condition caused by a symptomatic niche: Disorder, Syndrome, Disease?	67.7	No	93.5	Yes: included disorder	NA	NA
Which of the following terms would best cover the condition caused by a symptomatic niche? Niche or Cesarean Scar (Disorder/Disease/Syndrome)	61.3	No	54.8	No	96.2	Yes: included Cesarean Scar Disorder

Gynecological symptoms

Symptom	RoA in round 1(%)	Consensus reached in round 1	RoA in round 2 (%)	Consensus reached in round 2	RoA in round 3 (%)	Consensus reached in round 3:
Postmenstrual spotting<br>*Primary vs secondary symptom?	83.9	Yes: include	80.6	Yes: primary symptom	NA	NA
Dysmenorrhea	83.9	Yes: include	NA	NA	NA	NA
Chronic pelvic pain<br>*Primary vs secondary symptom?	83.9	Yes: include	87.1	Yes: secondary symptom	NA	NA
Abnormal uterine bleeding	83.9	Yes: include	NA	NA	NA	NA
Prolonged menses	83.9	Yes: include	NA	NA	NA	NA
Avoiding sexual intercourse<br>*Primary vs secondary symptom?	80.6	Yes: include	90.3	Yes: secondary symptom	NA	NA
Ovulation pain	-74,2	Yes: exclude	NA	NA	NA	NA
* Do you agree to combine pain dysmenorrhea and pain during spotting to pain during uterine bleeding?			71.0	Yes: include	NA	NA
Pain during spotting?	22.6	no	NA	NA	NA	NA

© 2023 Klein Meuleman SJM et al. *JAMA Network Open.*

*Pain during uterine bleeding<br>*Primary vs secondary symptom?	NA	NA	72.7<br>84.2	Yes: include<br>Yes: primary symptom	NA	NA
* Do you agree to combine spotting, prolonged menses and abnormal uterine bleeding to postmenstrual spotting?	NA	NA	71.0	Yes: include	NA	NA
Abnormal vaginal discharge<br>*Primary vs secondary symptom?	29.0	no	72.4<br>87.5	Yes: include<br>Yes: secondary symptom	NA	NA
Odor associated with abnormal uterine blood loss<br>*Primary vs secondary symptom?	48.4	no	73.0<br>83.4	Yes: include<br>Yes: secondary symptom	NA	NA
Dyspareunia<br>*Primary vs secondary symptom?	29.0	no	70.4<br>72.7	Yes: include<br>Yes: secondary symptom	NA	NA
Pollakisuria	-3.6	no	-71.4	Yes: exclude	NA	NA
Polyuria	-63.3	no	-86.7	Yes: exclude	NA	NA
Pain during micturition	-36.7	no	-87.1	Yes: exclude	NA	NA
Pain after urinating	-33.3	no	-93.3	Yes: exclude	NA	NA
**Fertility related symptoms**						
**Symptom**	**RoA in round 1(%)**	**Consensus reached in round 1**	**RoA in round 2 (%)**	**Consensus reached in round 2**	**RoA in round 3 (%)**	**Consensus reached in round 3:**
Secondary unexplained infertility<br>*Primary vs secondary symptom?	82.1	yes: include	70.0	Yes: secondary symptom	NA	NA
Secondary unexplained infertility despite ART<br>*Primary vs secondary symptom?	81.5	yes: include	72.4	Yes: secondary symptom	NA	NA
Technical issues with catheter during embryo transfer<br>*Primary vs secondary symptom?	80.0	yes: include	73.1	Yes: primary symptom	NA	NA
Secondary unexplained infertility combined with intrauterine fluid<br>*Primary vs secondary symptom?	67.9	no	92.9<br>70.4	Yes: include<br>Yes: primary symptom	NA	NA
Miscarriage	0.0	no			NA	NA
*More research is needed to conclude if miscarriages are a symptom of a uterine niche	NA	NA	96.7	Yes: include	NA	NA

Obstetrical symptoms

Symptom	RoA in round 1(%)	Consensus reached in round 1	RoA in round 2 (%)	Consensus reached in round 2	RoA in round 3 (%)	Consensus reached in round 3:
Cesarean scar pregnancy	71.0	Yes: include	NA	NA	NA	NA
Dehiscence of the uterus	73.3	Yes: include	NA	NA	NA	NA
Abnormal adhesive placenta	80.0	Yes: include	NA	NA	NA	NA
Uterine rupture	50.0	no	NA	NA	NA	NA
*Women with a obstetrical problem related to a uterine niche, have a complication of uterine niche disorder and not the disorder itself.	NA	NA	70.4	Yes: include	NA	NA

Social relationships and participation

Symptom	RoA in round 1(%)	Consensus reached in round 1	RoA in round 2 (%)	Consensus reached in round 2	RoA in round 3 (%)	Consensus reached in round 3:
Fatigue	-29.0	no	-87.1	Yes: exclude	NA	NA
Negative self-image<br>* Primary versus secondary symptom?	16.7	no	78.6<br>88.0	yes: include<br>yes: secondary symptom	NA	NA
Loneliness	-32.1	no	-100	Yes: exclude	NA	NA
Discomfort during leisure activities<br>*Primary vs secondary symptom?	22.5	no	70.4<br>77.3	yes: include<br>yes: secondary symptom	NA	NA
Depression	-10.3	no	-74.2	Yes: exclude	NA	NA

Categorization of symptoms

Question:	RoA in round 1(%)	Consensus reached in round 1	RoA in round 2 (%)	Consensus reached in round 2	RoA in round 3 (%)	Consensus reached in round 3:
** Do you agree with this categorization of symptoms?	NA	NA	NA	NA	93.1	Yes
** The proposed definition works for me, knowing that it will be evaluated and possible adjusted after 1 year.	NA	NA	NA	NA	77.8	Yes

© 2023 Klein Meuleman SJM et al. *JAMA Network Open.*

Diagnostic criteria

Question:	RoA in round 1(%)	Consensus reached in round 1	RoA in round 2 (%)	Consensus reached in round 2	RoA in round 3 (%)	Consensus reached in round 3:
The complaints of a symptomatic niche should start after a CS or should increase significantly after a CS.	80.6	yes: include	NA	NA	NA	NA
In order to call a group of symptoms a disease/disorder/syndrome, we need to define how to cure the disease. Do you think a patient can be cured from a symptomatic niche (this does not mean that all symptomatic niche should be treated)?	87.1	yes: include	NA	NA	NA	NA
It is obligatory that the complaints of a symptomatic niche should start after a CS.	64.5	no	96.8	yes: included	NA	NA
A patient need to be premenopausal to diagnose a symptomatic niche.	48.4	no	100	yes: included	NA	NA
Do you think there should be a minimal duration after a CS before you can make the diagnosis of a symptomatic niche?	41.9	no	NA	NA	NA	NA
*Minimal of three regular menstrual cycles after CS before diagnosis	NA	NA	93.5	yes: included	NA	NA

Conditions to exclude

Condition:	RoA in round 1(%)	Consensus reached in round 1	RoA in round 2 (%)	Consensus reached in round 2	RoA in round 3 (%)	Consensus reached in round 3:
Other uterine intracavitary pathology	93.5	yes: include	NA	NA	NA	NA
Other causes of postmenstrual spotting (such as continuous oral contraceptive use or IUD)	80.6	yes: include	NA	NA	NA	NA
Anovulatory cycles (due to endocrine disorders or breastfeeding)	29.0	no	100	yes: included	NA	NA
Endometriosis	16.1	no	-74.2	yes: excluded	NA	NA

© 2023 Klein Meuleman SJM et al. *JAMA Network Open.*

coagulation abnormalities	22.6	no	-87.1	yes: excluded	NA	NA
*Cervical dysplasia	NA	NA	74.2	yes: included	NA	NA
*Vaginal/uterine infections	NA	NA	74.2	yes: included	NA	NA
*Adenomyosis	NA	NA	-72.4	yes: excluded	NA	NA
*Uterine fibroids	NA	NA	-86.7	yes: excluded	NA	NA

Legend: * added in round 2, based on the feedback of previous round, ** added in round 3, based on the feedback in previous rounds.
Abbreviations: RoA, Rate of Agreement; CS, Cesarean section; ART, Assisted Reproductive Technology; IUD, intrauterine device

© 2023 Klein Meuleman SJM et al. *JAMA Network Open.*
