# Supplementary material for: Definition and Criteria for Diagnosing Cesarean Scar Disorder
Source: JAMA Netw Open. 2023 Mar 29;6(3):e235321. doi: 10.1001/jamanetworkopen.2023.5321 (PMC10061236; doi:10.1001/jamanetworkopen.2023.5321)
Supplement: Supplement 2. — Nonauthor Collaborators. The CSDi Study Group [file jamanetwopen-e235321-s002.pdf]

Supplemental Online Content: Nonauthor Collaborators
*First name, last name, and suffix (if applicable) are required and will appear in PubMed.

*Group Name(s): Cesarean Scar Disorder Study Group

***First Name and Middle Initial(s)**	***Last Name**	***Suffix (eg, Jr, III)**	Academic Degrees	Institution	Location (city, state/province, country)	Role or Contribution, eg, chair, principal investigator	Group (if more than 1 Group listed in the byline) and/or Subgroup (eg, Steering Committee)
Pere	Barri						
Tayfun	Cok						
Grigoris	Grimbizis						
Justin	Clark						
Samir	Helmy-Bader						
Davor	Jurkovic						
Attilio	di Spiezio Sardo						
Sandra	Tanahatoe						
Marcus	Rijken						
Dirk	Timmerman						
Frederic	Chantraine						
Ally	Murji						
Shuichiro	Tsuji						
Ilan	Timor						
Mary	Connor						
Andrea	Kaelin Agten						
Noel	Laure						
Wouter	Hehenkamp						
Roy	Mashiach						
Loïc	Sentilhes						
Michal	Promorski						
Osama	Naji						
Gwendolin	Manegold-Brauer						
Maddalena	Morlando						
Thierry	van den Bosch						
Sally	Collins						
Jian	Zhang						
Olivier	Donnez						

Supplemental Online Content: Nonauthor Collaborators

*First name, last name, and suffix (if applicable) are required and will appear in PubMed.

***First Name and Middle Initial(s)**	***Last Name**	***Suffix (eg, Jr, III)**	Academic Degrees	Institution	Location (city, state/province, country)	Role or Contribution, eg, chair, principal investigator	Group (if more than 1 Group listed in the byline) and/or Subgroup (eg, Steering Committee)
Shlomi	Cohen						
Judith	Huirne						
