# Supplementary material for: Definition and Criteria for Diagnosing Cesarean Scar Disorder
Source: JAMA Netw Open. 2023 Mar 29;6(3):e235321. doi: 10.1001/jamanetworkopen.2023.5321 (PMC10061236; doi:10.1001/jamanetworkopen.2023.5321)
Supplement: Supplement 3. — Data Sharing Statement [file jamanetwopen-e235321-s003.pdf]

Data Sharing Statement

Klein Meuleman. Definition and Criteria for Diagnosing Cesarean Scar Disorder. *JAMA Netw Open*. Published March 29, 2023. doi:10.1001/jamanetworkopen.2023.5321

Data
Data available: No

Additional Information
Explanation for why data not available: the Delphi round outcomes are already in the supplementary table
